# Supplementary material for: Expression of 3q Oncogene SEC62 Predicts Survival in Head and Neck Squamous Cell Carcinoma Patients Treated with Primary Chemoradiation
Source: Cancers (Basel). 2023 Dec 24;16(1):98. doi: 10.3390/cancers16010098 (PMC10778380; doi:10.3390/cancers16010098)
Supplement: Supplementary file 1 [file cancers-16-00098-s001.zip › cancers-2781595-supplementary.pdf]

Supplementary Table S1. Clinical and histopathological characteristics of SEC62 positive vs. SEC62 negative patients

	SEC62 positive	SEC62 negative
**Number of patients**	98	29
**Sex**		
male	78 (79.6%)	25 (86.2%)
female	20 (20.4%)	4 (13.8%)
**Tumor localization**		
Oral cavity	8 (8.2%)	0 (0%)
Oropharynx	45 (45.9%)	10 (34.5%)
Hypopharynx	23 (23.5%)	11 (37.9%)
Larynx	22 (22.4%)	8 (27.6%)
**T-Stage**		
T1	4 (4.1%)	0 (0%)
T2	15 (15.3%)	6 (20.7%)
T3	23 (23.5%)	7 (24.1%)
T4	56 (57.1%)	16 (55.2%)
**N-Stage**		
N0	18 (18.4%)	7 (24.2%)
N1	9 (9.2%)	3 (10.3%)
N2	66 (67.3%)	18 (62.1%)
N3	5 (5.1%)	1 (3.4%)
**M-Stage**		
M0	88 (89.8%)	29 (100%)
M1	10 (10.2%)	0 (0%)
**Grading**		
G1	2 (2%)	0 (0%)
G2	49 (50%)	16 (55.2%)
G3	47 (48%)	13 (44.8%)
**UICC-Stage**		
I	5 (5.1%)	1 (3.4%)
II	10 (10.2%)	6 (20.7%)
III	70 (71.4%)	22 (75,9%)
IV	13 (13.3%)	0 (0%)
**P16 expression**		
negative	78 (79.6%)	29 (100%)
positive	20 (20.4%)	0 (0%)
